# Missed Foreign Body Ingestion in a Cognitively Impaired Adult: A Case Report

**DOI:** 10.7759/cureus.87157

**Published:** 2025-07-02

**Authors:** Zorab Rahim, Adnan Rahman

**Affiliations:** 1 General Medicine, Aneurin Bevan University Health Board, Cwmbran, GBR; 2 Gastroenterology, Aneurin Bevan University Health Board, Cwmbran, GBR

**Keywords:** cognitive impairment and dementia, deliberate foreign body ingestion, missed diagnosis, timing of endoscopy, upper endoscopy

## Abstract

The diagnosis of foreign body ingestion is often clear from the history; diagnostic difficulty and delay in management arise when patients and carers are unable to give a clear history of ingestion. We present the case of a cognitively impaired adult who was misdiagnosed with gastritis when they had, in fact, been suffering from vomiting secondary to foreign bodies impacted in the oesophagus. Timely endoscopy in accordance with the European Society of Gastrointestinal Endoscopy (ESGE) guidelines prevented the potentially significant complications associated with foreign body ingestion.

## Introduction

Foreign body ingestion is a commonly encountered acute presentation, but difficulties in diagnosis are encountered in adults with cognitive impairment who are unable to provide a history, which can lead to delays in investigation and treatment.

Risks of undiagnosed foreign body ingestion include laceration, obstruction, perforation and fistula formation [1]. 

Whereas the link between psychiatric illness and foreign body ingestion is clear [2], data on the incidence of foreign body ingestion in cognitively impaired adults is mostly limited to case reports, highlighting a clear gap in the literature [3,4]. A 2023 Australian multicentre study of deliberate ingestions found 87% of episodes had suicidal or parasuicidal intent and 14% of patients had intellectual disabilities [5]. 

In adults, most ingested objects pass spontaneously; however, 10-20% require endoscopic intervention, and less than 1% require surgery due to the complications mentioned [6]. Nevertheless, the probability of both minor and significant adverse events increases with the duration of impaction. A case series of 241 patients comparing outcomes of patients undergoing endoscopic intervention for foreign body ingestion before and after 24 hours showed higher rates of oesophageal ulceration (21.1 vs. 7.2%) in those undergoing endoscopy 24 hours after ingestion [7], highlighting the deleterious effects of missed diagnosis in foreign body ingestion. 

We present the case of a patient with profound alcohol-related brain damage who re-presented to the hospital with vomiting due to a previously missed foreign body ingestion.

## Case presentation

A 54-year-old lady with severe cognitive impairment was referred to the acute medical unit from her residential home with persistent vomiting not responding to anti-emetics. Her only significant past medical history was alcohol-related brain damage (ARBD), which was so severe that the patient required supervision by carers on a one-to-one basis. As a result of her brain damage, the patient was completely non-verbal and therefore unable to give a history.

A collateral history from the patient’s carer revealed that the patient had been suffering from frequent hourly vomiting, exacerbated by eating over the past two weeks. The content of the vomit was described as clear and intermittently bilious. There was no access to alcohol in the home and no history of smoking. The patient had been discharged a few days earlier from a peripheral acute medical unit with a presumed diagnosis of gastritis.

Initial observations and baseline blood tests were significant only for a heart rate of 101 BPM and C-reactive protein of 18 mg/L. Other observations and bloodwork, including full blood count, urea and electrolytes and liver function tests, were all within normal limits. 

**Figure 1:**
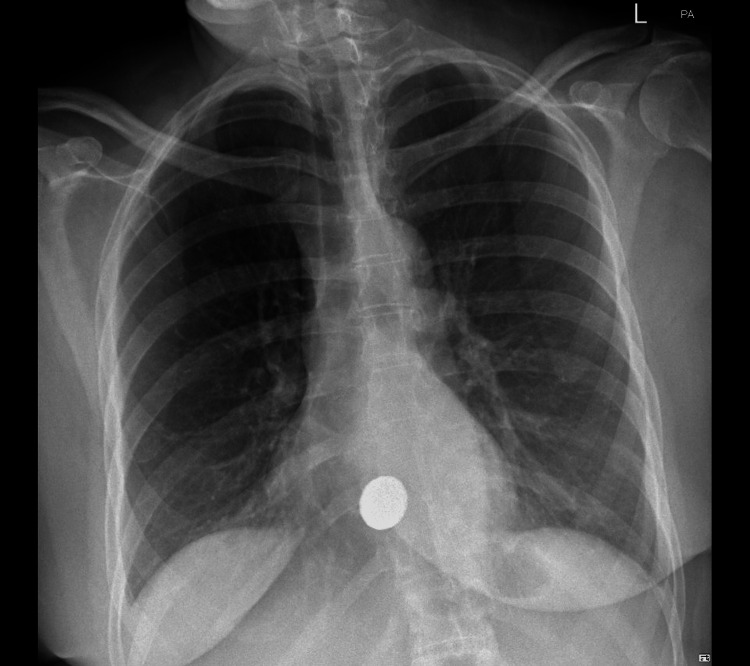
Rounded metallic density lodged in the lower oesophagus

On this admission, given the frequency and severity of the vomiting and risk of oesophageal perforation, a chest X-ray was ordered (Figure 1). There was no subcutaneous emphysema seen; however, a rounded metallic density projected over the midline just above the hemidiaphragms showed the cause of the patient’s symptoms. On further questioning of her carer, the patient did have a history of swallowing foreign bodies; however, there was no eyewitness account of this on this occasion. 

Oesophagogastroduodenoscopy (OGD) was performed 17 hours after presentation in theatres under propofol sedation with anaesthetic support. On endoscopic examination, two £1 coins were identified lodged together in the mid to lower oesophagus, surrounded by food debris. The appearance suggested they had been impacted for a prolonged period. Due to their positioning, the coins were removed individually using grasping forceps. 

Following removal, a post-extraction oesophagogastroduodenoscopy (OGD) was performed. No obstructing pathology was identified. However, there were superficial linear erosions and moderate oesophagitis observed between 30 and 35 cm from the incisors, corresponding to the site of impaction. The remainder of the upper gastrointestinal tract appeared normal on examination, and no further follow-up or treatment was required. 

The patient’s symptoms promptly resolved after the extraction of the two £1 coins, and she was discharged the next day once she was able to tolerate her lunchtime meal. 

## Discussion

This case highlights the importance of maintaining a low threshold for investigating foreign body ingestion in patients who are unable to give a reliable history and present with symptoms of retching, vomiting, refusal to eat and choking [8]. This is especially so as cognitive impairment is a risk factor for foreign body ingestion [9].

As seen with our patient, it is possible that foreign body ingestion may have an atypical presentation that includes behavioural change or agitation. The lack of specificity of these signs may contribute to diagnostic overshadowing, whereby these symptoms are attributed to an exacerbation of the patient’s existing mental health problems rather than as a sign of a new physical health problem, such as ingestion of a foreign body. 

The importance of obtaining a collateral history cannot be stressed enough. Residential home staff, carers and family members should be consulted when there is a suspicion or diagnostic uncertainty regarding a cognitively impaired patient’s symptoms, especially in the context of a potential foreign body ingestion. Particular attention should be given towards the likely nature of the object swallowed and whether it carries any high-risk features, as this will determine the timing of endoscopy.

In cases of complete oesophageal obstruction, or when sharp or pointed items or batteries or magnets are lodged in the oesophagus, the European Society of Gastrointestinal Endoscopy (ESGE) recommends emergent OGD, ideally within 2 hours and no later than 6 hours. For other oesophageal foreign bodies, such as coins, that do not cause total obstruction, an urgent OGD should be performed within 24 hours [10]. 

ESGE also advises the use of plain radiographs to help determine the presence, location, size, shape, and number of ingested foreign bodies, particularly when the object is radiopaque or its nature is uncertain [10]. 

It is worth noting that a normal X-ray does not rule out an ingestion, sometimes hindering diagnosis in the cognitively impaired. Radiolucent foreign bodies that are commonly missed on plain film include fish bones, wood, plastic, glass and thin metal objects [8]. If suspicion of foreign body ingestion remains high, definitive imaging (CT) or diagnostic endoscopy should be considered [11,12]. 

## Conclusions

A high index of suspicion for foreign body ingestion is required in the vomiting, cognitively impaired patient. Plain radiographs are a low-cost and widely available first-line imaging modality that can detect most metallic foreign bodies. Clinicians should obtain a collateral history in cases where a patient is unable to reliably provide a history of ingestion themselves. Clinicians should also recognise that cognitively impaired patients who have ingested a foreign body may present with non-specific signs and symptoms which should not be mistakenly attributed to an exacerbation of previous mental health problems. Combining these recommendations with timely endoscopy in line with local guidelines is key to preventing serious complications that can result from prolonged impaction of a foreign body within the gastrointestinal tract. 
